# Bisphosphonates and Atypical Fractures of Femur

**DOI:** 10.4061/2011/754972

**Published:** 2011-03-31

**Authors:** Tero Yli-Kyyny

**Affiliations:** Department of Orthopedics, Traumatology and Hand Surgery, Kuopio University Hospital, Puijonlaaksontie 2, P.O. Box 1777, 70211 Kuopio, Finland

## Abstract

Bisphosphonates are the most widely prescribed medicines for the treatment of osteoporosis and have generally been regarded as well-tolerated and safe drugs. Since 2005, there have been numerous case reports about atypical fractures of the femur linked to long-term treatment of osteoporosis with bisphosphonates. Some attempts to characterize pathophysiology and epidemiology of these fractures have been published as well. However, as the American Society for Bone and Mineral Research (ASBMR) concluded in their task force report, the subject warrants further studies.

## 1. Introduction

Bisphosphonates are widely used for treatment of osteoporosis. It has been shown in the randomized clinical studies that bisphosphonates reduce the incidence of osteoporotic vertebral and hip fractures when used for the treatment of postmenopausal osteoporosis [1–4]. Bisphosphonates act by inhibiting the osteoclast function and inducing osteoclast apoptosis. This leads to suppression of bone resorption and increase of bone mineral density. Based on studies on experimental animals, it has been shown, however, that suppression of bone resorption might lead to suppression of bone turnover and creation of hard, but more brittle bone [5, 6].

The optimal duration of the bisphosphonate therapy remains also a question. Based on current evidence, the continuing of bisphosphonate therapy beyond five years does not seem to lower osteoporotic fracture risk as much as the first years of therapy [7].

In 2005, Odvina et al. published a case series about nine osteoporotic patients on long-term bisphosphonate therapy who had acquired atypical fractures [8]. In their report, suppressed bone turnover by bisphosphonates was suggested as etiologic factor for the fractures. 

Since the publishing of this report, a few case reports about the subject have been published. The American Society for Bone and Mineral Research also has published recently a task force report about atypical femoral fractures [9] as well as two international osteoporosis societies [10]. Some review articles about the subject have also been published recently [11, 12]. In this paper, published case series and reports are reviewed as well as current knowledge about the epidemiology and pathophysiology of these fractures.

## 2. Published Case Reports and Case Series

The original case series of Odvina et al. [8] consisted of nine patients having fractures in various nonvertebral sites, actually only three patients had fractures of femoral shaft, while two other patients had fractures of proximal femur. The patients in this original case series were well studied; the case series included histomorphometric data, biomechanical markers of bone turnover and bone densitometric measurements. They noted that in the histomorphometric samples a severely suppressed bone turnover was seen. It also was suggested that use of estrogens or corticosteroids could be predisposing factors for these fractures, since three of the patients were on estrogen therapy and two of them had used corticosteroids.

**Figure 1:**
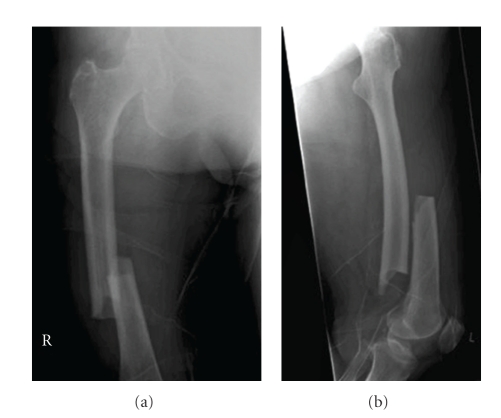
A case of bilateral femur fractures in a patient who had been on alendronate therapy for six years.

After the original report by Odvina et al., a number of case reports have been published [13–29]. In the case series by Kwek et al. [18] the major radiologic features of these fractures were defined as (a) cortical thickening in the lateral (tension) side of the subtrochanteric region, (b) transverse fracture, and (c) medial cortical spike (Figure 1). These features were included also in the ASBMR definition of atypical femoral fractures. In the ASBMR task force report the atypical fractures were, however, also required to be associated with no trauma or minimal trauma and to be noncomminuted.

The original case series by Odvina et al. included two patients with bilateral fractures of femoral shaft. Subsequent case reports and case series have included both unilateral [13–16, 18, 20, 25] and bilateral cases [16, 17, 25–29]. It has been demonstrated that patients having unilateral atypical femoral fractures often have stress injury-like cortical hypertrophy in their contralateral limb [14], suggesting that bisphosphonate effect on these patients' femurs most likely is bilateral. In the series by Kwek et al. [18], the incidence of contralateral cortical hypertrophy was estimated to be 53%.

Another characteristic feature of atypical femoral fractures are prodromal symptoms. Goh et al. [14] were the first authors reporting of ipsilateral prodromal pain before subsequent fracture. In their series of nine alendronate-treated patients with atypical subtrochanteric fractures, five patients had experienced pain or discomfort in the fractured limb, between two to six months before the injury. The prodromal symptoms and cortical hypertrophy were correlated with radiologic cortical stress reaction by Koh et al. in their retrospective series of 16 patients [30]. They identified a “dreaded black line”, a radiologic finding known to be associated with nonunion of stress fractures [31], in four patients' X-rays taken because of prodromal symptoms before the fracture. They suggested that the presence of the dreaded black line in a symptomatic patient should indicate prophylactic nailing, since in their retrospective series it seemed to herald a complete fracture.

The case reports and case series published so far have mostly included patients on alendronate therapy [13–20, 25–29]. There are, however, reports about patients with atypical fractures who have been on pamidronate [21] or risedronate [22–25] as well. The greater amount of cases linked to alendronate than other bisphosphonates is most probably a consequence of alendronate's position as the most frequently used bisphosphonate for (postmenopausal) osteoporosis.

In the published case series and reports it has been pointed out that concomitant use of other pharmacologic agents with alendronate could be a predisposing factor for atypical femoral fractures. In the original Odvina's report [8], coadministration of estrogen and glucocorticoids were raised as potential predisposing factors. In subsequent reports frequent use of these agents has also been noted as well as use of oral proton-pump inhibitors [16, 21, 27, 28]. Giusti et al. in their systematic review estimated the concomitant use of oral glucocorticoids to be 25.5%, estrogens 11.8%, and proton pump inhibitors 38.9% in patients with atypical fractures. There is a good number of published cases of patients not using any of the above-mentioned agents [14, 18], however, and therefore a direct causal relationship cannot be established. Moreover, the use of the above-mentioned agents may well be frequent in osteoporotic patients. Glucocorticoid use is a well-known cause of secondary osteoporosis, estrogens are often used by postmenopausal women, and bisphosphonates cause dyspeptic symptoms that may lead to use of proton pump inhibitors.

## 3. Pathophysiology

In the original case series by Odvina et al. [8], an iliac crest bone biopsy was obtained from all nine patients. All of their patients showed a severe depression of bone formation with absence of double-tetracycline labelling. The case reports and case series reviewed in this paper include data of iliac crest bone biopsies after double-tetracycline labeling from 19 patients [8, 16, 20, 22, 26, 29]. Of them, 18 have shown severe depression of bone turnover (defined as complete lack of tetracycline labels). 

It is known that long-term bisphosphonate use suppresses bone turnover, but in the previous histomorphometric studies single or double tetracycline labels have almost always been detected, contrary to findings in patients with atypical fractures [32, 33]. Also, Bone et al. found that even combination therapy of alendronate and estrogen did not result in complete disappearance of tetracycline labels [34]. The samples in these studies were, however, collected from patients treated for 2-3 years with bisphosphonates. There are some studies [35, 36] where the authors have collected histomorphometric samples from patients treated with alendronate for 5-6 years in average. In both of these studies, the bone turnover rate was clearly lowered and in the study of Chapurlat et al. double-tetracycline labels were lacking in 1/3 of patients [36]. In light of these findings, it seems prudent to state that long-term (over five years) therapy with bisphosphonates may lead to a severe suppression of bone turnover that in itself may be a predisposing factor for the atypical fractures of femur.

The safety of long-term bisphosphonate therapy has been questioned earlier on the basis of experimental studies. Mashiba et al. [5] treated beagle dogs with alendronate or risedronate for one year and observed a suppression of bone turnover and increase of microdamage accumulation in dogs' ribs. The doses used were five times greater than clinical doses used in humans for treatment of osteoporosis. Same phenomenon was observed in beagles' vertebrae and femoral necks [6]. There was no difference between alendronate and risedronate in the microdamage accumulation suggesting that possible adverse effects of bisphosphonates on bone should not be restricted to alendronate only. 

Bone turnover in patients with atypical fractures has also been estimated with the help of biochemical markers (urinary excretion of N-telopeptide and/or hydroxyproline) in some of the case reports and series mentioned above [8, 16, 20, 25–27]. The results from these studies are inconclusive. There are reports where the markers of bone turnover have indicated decreased turnover [16] and increased turnover [26]. However, in most reports, the biochemical markers have been within the reference limits [8, 20, 25, 27]. This most probably reflects the fact that biochemical markers have poor sensitivity and specificity for detection of low bone turnover. Similarly, the bone mineral density data of the published cases does not provide any conclusive evidence of the pathophysiology of the atypical fractures of femur.

## 4. Epidemiology

There is little epidemiologic data concerning the atypical fractures of femur in bisphosphonate users so far. There have been some attempts to determine the incidence of these fractures and to find out whether long-term bisphosphonate use might lead to rising incidence of subtrochanteric/diaphyseal femoral fractures.

Schilcher and Aspenberg identified the incidence of atypical fractures in the area of two healthcare districts in Sweden [37]. With the help of national drug delivery register, they were able to find out the prevalence of bisphosphonate use in their area. They were able to find out 8 cases where fracture configuration was consistent with the radiologic appearance of atypical fractures. Of these patients, five were bisphosphonate users. Using these findings they estimated the incidence of atypical fractures in patients on continuous bisphosphonate therapy to be 1/1000 per year (CI: 0.3–2).

Black et al. performed secondary analyses of three large randomized, controlled bisphosphonate trials [38]. Among 14,195 women they were able to find 12 fractures in 10 patients. The fractures were classified as occurring in the subtrochanteric or diaphyseal femur. A weakness of the study was the lack of radiographs of all the patients. They estimated the rate of subtrochanteric or diaphyseal fractures of femur to be 2.3 per 10,000 patient-years. The relative hazard rates for alendronate (FIT trial) was 1.03; for zoledronic acid (HORIZON-PFT trial) 1.50 and for continued alendronate use (FLEX trial) 1.33. The confidence intervals were wide, probably due to the fact that none of these trials was originally powered to detect a relatively uncommon side-effect of the bisphosphonates.

Another method to estimate the occurrence of subtrochanteric or diaphyseal femur fractures in bisphosphonate-treated patients was utilized by Abrahamsen et al. [39]. Their study included a cross-sectional and matched control cohort studies. In the cross-sectional part they were able to find out that the same percentage (7%) of subtrochanteric fracture patients were alendronate users as of the patients with hip fractures. In the cohort study they found out that alendronate use carried a similar risk for subtrochanteric or diaphyseal femur fractures (HR = 1.64) than for hip fractures (HR = 1.50). Conclusion of the investigators was that the risk for the proximal femoral fractures was caused by the osteoporosis itself, since the risk was similar for both these fractures. The limitations of the study include the lack of analysis of the radiographs. The fracture groups included also high-energy patients. 

Vestergaard et al. studied the incidence of subtrochanteric and diaphyseal femoral fractures both before and after the start of various drugs against osteoporosis [40]. Their study was based on hospital discharge registers and included age- and gender-matched controls. They were not able to find an increased incidence of the above-mentioned fractures among 103,562 patients receiving drugs against osteoporosis. Although the atypical fractures were not specifically identified since no X-rays were analyzed, the study supports the conclusion that the skeletal benefits of antiresorptive agents outweigh the possible skeletal side-effects of bisphosphonates.

We have also made an estimate of the incidence of atypical fractures of femur in our university hospital's catchment area using methods similar to Schilcher and Aspenberg (unpublished data). Interestingly, we resulted in an estimate of incidence of 0.5/1,000 patient-years among bisphosphonate-users, not very far from the estimate by Schilcher and Aspenberg.

## 5. Conclusions

Based on published case reports and case series, the atypical fractures of femur seem to occur rarely without a long-term treatment of osteoporosis with bisphosphonates. Since the evidence-base for osteoporosis treatment with bisphosphonates for more than five years is relatively thin, it seems prudent to consider the benefits and risks on a patient who has received the treatment for more than five years. However, as it is not yet known whether the risk of adverse effects of medication can be avoided by drug holidays or by discontinuing the bisphosphonate therapy, more research is clearly needed. The atypical femoral fractures with their possible prodromal symptoms should be remembered on any patient on bisphosphonate therapy, especially if they are receiving therapy with estrogens, oral glucocorticoids, or proton pump inhibitors.

Based on the available data, the most likely pathophysiologic mechanism leading to these fractures seems to be the suppression of bone turnover by bisphosphonates. However, more research in this regard is needed as well.

Finally, the true incidence and epidemiology of this possible adverse effect of osteoporosis treatment is very poorly known. Therefore, it is very easy to agree with ASBMR, calling for international registry for cases of atypical femoral fractures. The guidelines suggested for the future research can be agreed as well in order to be better able to characterize these fractures. 

While there is concern that there may be more atypical femoral fractures in future, when more osteoporotic patients have received bisphosphonate therapy for more than five years, there is not yet any evidence which would urge physicians to discontinue the therapy, at least before five years. In the light of current knowledge, the positive effects of bisphosphonates outweigh their adverse effects.
